# Implementation of the Austrian Nursing Minimum Data Set (NMDS-AT): A Feasibility Study

**DOI:** 10.1186/s12911-015-0198-7

**Published:** 2015-09-17

**Authors:** Renate Ranegger, Werner O. Hackl, Elske Ammenwerth

**Affiliations:** 1 Steiermärkische Krankenanstaltengesellschaft m.b.H., Management/Pflege, Stiftingtalstraße 4-6, 8010 Graz, Austria; 2 UMIT - University for Health Sciences, Medical Informatics and Technology, Institute of Biomedical Informatics, Eduard Wallnöfer-Zentrum I, 6060 Hall in Tirol, Austria

**Keywords:** Nursing care, Nursing process, Nursing informatics, Nursing records, Nursing classification, Decision making, Data collection, Healthcare data, Nursing data, Austria

## Abstract

**Background:**

An Austrian Nursing Minimum Data Set (NMDS-AT) has been developed to describe the diversity of patient populations and variability of nursing care based on nursing diagnoses, nursing interventions, and nursing outcomes. The aim of this study is to test the feasibility of using this NMDS-AT by assessing the availability of data needed for the NMDS-AT in routine nursing documentation, and to assess its reliability and usefulness.

**Methods:**

Data were collected in a general hospital from patient records of 20 patients representing 457 patient days. Availability of needed data was assessed by two raters in a chart review based on an NMDS-AT form. The interrater reliability (*n* = 20) and intrarater reliability (*n* = 5) was assessed using Cohen’s kappa coefficient and intraclass correlation coefficient (ICC). Usefulness was assessed by verifying whether typical analysis questions can be answered by the documented NMDS-AT data.

**Results:**

In the 20 patient records, thirteen nursing diagnoses, 50 nursing interventions, and five nursing outcomes occurred, representing 68 (58.6 %) of the overall 116 data elements of the NMDS-AT. The data were found at different data sources (e.g., electronic nursing record or paper-based fever chart) and in various forms (e.g., standardized or free text).

The interrater reliability of the thirteen nursing diagnoses showed kappa values (percentage of agreement) ranging from 0.35 (85 %) to 1.00 (100 %). The 50 nursing interventions showed ICCs ranging from 0.03 to 1.00. All nursing outcomes showed an ICC of 1.00. The intrarater reliability showed 100 % agreement. Performing typical analysis questions showed that the extracted NMDS-AT data are able to answer questions of clinical management, of policy makers, and of nursing science.

**Conclusions:**

The NMDS-AT was found to be feasible: needed data was available in the analysed patient records, data extraction showed good reliability, and typical analysis could be performed and showed interesting results. Before the NMDS-AT can be introduced in healthcare institutions, the following challenges need to be addressed: 1. improve the quality of nursing documentation; 2. reduce fragmentation of documentation; 3. use a standardized nursing classification system; and 4. establish mappings between nursing classification systems and the NMDS-AT.

## Background

Considering the sustained, increasing pressure on health expenditure – characterized by ageing populations, rising public expectations, and the introduction of new technology – several European countries have been implementing a wide range of cost containment and quality assurance strategies, based on available healthcare data. The most common sources of healthcare data are medical registries (such as cancer registries), administrative and billing data, population surveys, or patient surveys [1]. Data reflecting efficiency and quality of nursing care is usually not available.

Nursing Minimum Data Sets (NMDS) have been proposed to systematically describe nursing care. Already in 1988, Werley and Lang stressed the need for an NMDS that describes nursing in terms of nursing diagnoses, nursing interventions, nursing outcomes, and nursing intensity [2]. Nursing Minimum Data Sets have been defined as ‘a systematic registration of the smallest possible number of unequivocally coded data, with respect to or for the purpose of nursing practice, making information available to the largest possible group of users according to a broad range of information requirement’ [3]. An NMDS may provide the following benefits: access to comparable nursing care data on a local, regional, national, and international level [4]; description of nursing care in different populations and variety of settings; availability of data for research activities; evaluation of costs and outcomes of nursing care; benchmarking of nursing quality indicators; extrapolation of trends in nursing care; and allocation of resources of hospitals [5, 6]. An NMDS aims at supporting nursing managers, health policy decision makers, public health experts, and nursing researchers [4].

In 1991, Werley et al. established an NMDS in the United States (US-NMDS). This was the first attempt to standardize the collection of essential nursing data for the comparison of nursing data across populations, settings, geographic areas, and time [5].

Belgium established its own Nursing Minimum Data Set (B-NMDS) in 1988. It is still the country in the world with the largest NMDS usage at a national level [7]. Data for the B-NMDS is collected in all Belgian hospitals and is used by hospital managers to support staffing decisions and by the Ministry of Health to allocate hospital financing [4, 8]. In 2007, the original B-NMDS was replaced by a renewed data set based on the Nursing Intervention Classification (NIC), leading to B-NMDS II [9].

In Germany, a research project was carried out in 2006 in order to investigate the transfer of the B-NMDS II to German hospitals (G-NMDS) [10]. NMDS developments are also in progress in Australia, Canada, and in European countries such as Finland or the Netherlands [11, 12]. Furthermore, a project to develop an international Nursing Minimum Data Set (iNMDS) was started in 2001, co-sponsored by the International Council of Nurses (ICN) and the International Medical Informatics Association, Nursing Informatics Special Interest Group (IMIA NI-SIG). This project focused on coordinating international nursing data to describe nursing care around the world [13]. In 2010, a study about the application of the iNMDS was published [14]. This was the latest publication about developments of the iNMDS.

In Austria, there is currently no systematic, national-level collection of nursing care data, and no Austrian NMDS exists. Moreover, information about nursing practice is missing in available regional and national healthcare databases [15]. Yet Austria has one advantage compared to other countries: by law, it is mandatory to document all steps of the nursing process; thus it is mandatory for nurses to document nursing diagnoses, nursing interventions, and nursing outcomes in the patient record (GuKG, 1997) [16]. For all inpatients, therefore, the information that will be a core part of an NMDS is available, mostly in structured form.

Since 2012, a research project has been underway to develop a Nursing Minimum Data Set for Austria (NMDS-AT). As a first step, the available NMDS of other countries were reviewed to identify typical data elements and associated objectives [17]. Thereafter, a three-round Delphi method with national nursing experts was conducted to identify possible core data elements for an Austrian NMDS [18, 19].

The proposed NMDS-AT has a general inpatient focus and the included elements are comparable to other NMDS. For example, the NMDS-AT includes patient problems comparable to the nursing phenomena of the NMDS of the Netherlands [12]. The nursing interventions are comparable to the German NMDS and to the B-NMDS II [10]. The NMDS-AT includes 33 nursing diagnoses, 6 nursing outcomes, and 78 nursing interventions [19]. Some of the data elements in the NMDS-AT are similar to corresponding definitions in relevant classification systems, e.g., the North American Nursing Diagnosis Association International (NANDA-I) [20] or the Nursing Intervention Classification (NIC) [21, 22]. For the nursing outcomes, nursing sensitive outcomes comparable to the American Nurses Association quality indicators [23] were used.

The NMDS-AT has been published [19]; however, it has not yet been used in nursing practice to extract data from routine nursing documentation. The objective of this pilot study is to investigate the feasibility of using the NMDS-AT to extract data from routine documentation. Feasibility comprises whether data is available in routine documentation and can be reliably extracted to the NMDS-AT, and whether the extracted data is indeed useful for answering typical analysis questions for clinical management, policy makers, and nursing researchers. The aim of this study is thus:

- to examine whether the core data elements of the NMDS-AT are available in a typical routine nursing documentation system of an Austrian hospital,
- to assess interrater reliability and intrarater reliability when extracting NMDS-AT data from routine nursing documentation, and
- to gain insight into the usefulness of the data from the NMDS-AT, by verifying whether typical analysis questions can be answered by the documented NMDS-AT data.

## Methods

### Design

A chart review of 20 patients of an Austrian hospital was conducted, and data on patient problems, nursing interventions, and nursing outcomes were extracted from routine nursing documentation into the NMDS-AT by two raters. The study took place between December 2014 and March 2015.

### Setting and sample

The study was conducted in an acute care hospital in the Austrian state of Styria, with a capacity of around 100 beds. Two adult general units were included in this study. Ward 1 is a rehabilitation and aftercare ward; Ward 2 is a rehabilitation ward with a focus on geriatrics. The hospital was selected based on the following three selection criteria: first, there are medical as well as surgical patients on these units; second, the units regularly applied nursing screenings and assessment instruments; third, the units treat ‘long-stay’ patients, with an average length of stay of around 20 days. It was assumed that for these patients a comprehensive and detailed nursing documentation would be available, allowing a good feasibility test of the NMDS-AT.

The hospital uses an electronic nursing documentation system and a paper-based medical record, the latter comprising in particular the fever chart. The electronic nursing record comprises forms to document patient assessment, nursing diagnosis, nursing goals, nursing interventions, nursing reports, and nursing discharge letter, as well as forms to ascertain and record special nursing care, such as the Barthel Index of Activities of Daily Living, Nutritional Risk Screening, and documentation of patient falls. Wound management is usually treated in an interdisciplinary manner, and the professionals commonly use a specific electronic wound care form for this activity or document it on the paper-based fever chart. The paper-based fever chart also includes medical reports, vital signs, medications, and other interventions ordered by physicians.

Medical diagnoses are coded in ICD-10 and are used for billing based on the diagnosis-related groups (DRG); in Austria, this standardized medical documentation is also called ‘Minimum Basic Data Set’ (MBDS) [24].

For classification of nursing diagnoses, nursing interventions, and nursing outcomes, locally developed classification systems of the Styrian Hospital Organisation (KAGes) are used.

A random sample of 20 patients (corresponding to 457 patient days) was selected from a list of 54 discharged patients during the study period. To assess test-retest reliability, five randomly selected patient charts were chosen. To ensure patient privacy, the used data were extracted from anonymous patient records; submission to the Federal Act concerning the Protection of Personal Data in Austria was therefore not necessary under § 46 (allowing scientific research and statistics of available data under certain circumstances) [25]. The study received ethical approval from the Medical University of Graz (reference number: 25–541 ex 12/13).

### Data extraction

**Fig. 1:**
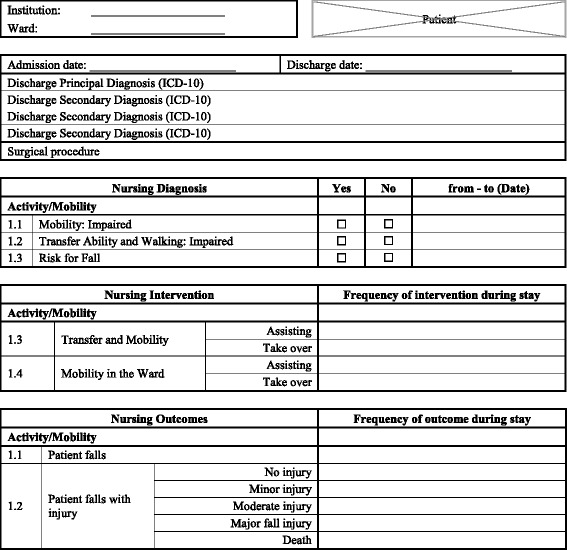
Examples of data elements from the NMDS-AT data extraction form. Three of the 33 nursing diagnosis, two of the 78 nursing interventions, and two of the five nursing outcomes are presented. The full form (in German) and the instruction manual can be request from the author

For data extraction from the patient chart, a standardized form and an instruction was prepared (see Fig. 1), describing how to map data from the routine documentation to the NMDS-AT. A member of the research team (RR) and a nursing staff member were recruited as raters. Both raters have worked in direct patient care and are experts in nursing documentation. A training session was held to train in the use of the NMDS-AT.

The data were extracted independently by both raters, using information from the electronic nursing documentation and from the paper-based fever chart of each patient. After three months, in March 2015, one rater (RR) repeated the chart review for five randomly selected patients in order to assess test-retest reliability.

Data were extracted for the chosen patients from the day they were admitted to the day of discharge.

### Standardized form for data extraction

To extract the NMDS-AT data from each patient record, a form was developed, together with an accompanying instruction manual. This form contained the 33 nursing diagnoses, 6 nursing outcomes, and 78 nursing interventions contained in the NMDS-AT. In addition, the hospital name, patient demographics, and selected medical care elements (such as medical diagnosis, surgical procedures) were also extracted.

The data elements are a mix of dichotomous and ratio variables. The occurrence of a nursing diagnosis was e.g., described by ‘yes/no’ categories. For nursing interventions and nursing outcomes, the frequency of occurrence was counted per day. Figure 1 shows examples of data elements from the NMDS-AT data extraction form.

All the data elements in the NMDS-AT were defined in an instruction manual, which was used as a guideline to extract the data. It also contained definitions of each data element, such as the descriptions of the different categories of fall-related injury [23].

### Data analysis

Statistical analysis was performed using SPSS 20 [26].

To assess data availability, a review was conducted for each item of the NMDS-AT to see whether it could be found in the 20 patient records.

For each data item, the form and location of routine documentation (electronic or paper-based documentation, standardized or free-text) was also documented.

To assess reliability, the interrater reliability between both raters and the intrarater reliability (test-retest reliability), using Cohen’s kappa coefficient for nominal data elements [27] and interclass correlation coefficient (ICC) for the ratio data elements [27, 28], were calculated. According to [29], a Kappa ($\widehat{\kappa}$) of (0.61 ≤ $\widehat{\kappa}$ ≤0.8) was considered ‘substantial’ , a kappa of (0.81 ≤ $\widehat{\kappa}$ ≤1.0) ‘almost perfect’. An interclass correlation (ICC) of >0.75 was considered ‘excellent’ [30].

To assess usefulness, we identified typical NMDS analysis questions from the literature [17, 31]. We then performed these data analysis on the extracted NMDS-AT data. These data analysis questions reflected typical analysis questions for an NMDS by clinical managers, policy makers, and researchers, namely:

1. Is it possible to describe the diversity of patient populations and the variability of nursing care?
2. Is it possible to measure nursing care intensity (nursing workload) to support human resource planning and support the distribution of funds?
3. Is it possible to illustrate nursing’s contribution to patient care to provide arguments for healthcare decision makers?
4. Is it possible to evaluate nursing outcomes to support quality management and to improve patient safety, and to identify evidence-based ‘best practice’?
5. Is it possible to describe nursing care for benchmark activities?
6. Is it possible to report frequencies over long time periods to show trends in nursing care?

To analyse these six different analysis questions, selected statistical analysis of the extracted data was performed, based on a patient day level [31]. The chosen statistical analysis refers to all 457 patient days (20 patients). To test questions 1 and 2, aggregated nursing diagnoses (nursing diagnosis domains) and aggregated nursing interventions (nursing intervention domains) were analysed by using mean percentage frequencies based on a patient day level. Question 3 was analysed by using the mean score of the Barthel Index of Activities of Daily Living at admission and discharge. To assess question 4, the mean percentage frequencies of three essential nursing interventions regarding prevention of pressure ulcers were compared with the nursing outcome ‘pressure ulcer incidence’. Questions 5 and 6 were evaluated using a combination of the aforementioned analysis methods.

## Results

Overall, data of 457 ‘patient days’ were extracted for the 20 included patients by both raters. The time needed to manually extract the data from the patient records varied from 20 to 45 min per patient.

### Sample characteristics

The mean age of the included 20 patients was 76 years (range 55 – 92 years, SD ±10). Fourteen patients were female. The average length of stay of the 20 patients was 23 days (range 2 – 49 days, SD ±11). Nine patients from the two wards had an internal medical disease; eleven patients had undergone a surgical procedure. The predominant medical diagnoses were orthopaedic procedures (*n* = 10), musculoskeletal system diseases (*n* = 4), cardiac diseases (*n* = 2), skin diseases (*n* = 2), an abdominal operation (*n* = 1), and a malignant disease (*n* = 1). An overview of sample characteristics regarding both wards is illustrated in Table 1.

**Table 1:** Sample characteristics of Ward 1 (rehabilitation/aftercare) and Ward 2 (rehabilitation/geriatrics)

	Ward 1 (*n* = 10)	Ward 2 (*n* = 10)
Age (years)	73 (SD ±9)	77 (SD ±10)
Female	6	8
Male	4	2
Length of inpatient stay	25 (SD ±15)	21 (SD ±7)
Surgical procedure	6	5

### Availability of data

Patient demographics data, such as sex, age, or admission and discharge date, and medical diagnosis are already included in the Austrian Minimum Basic Data Set and are thus electronically available.

In this study, the main focus was on availability of data representing nursing care. Thirteen nursing diagnoses, 50 nursing interventions, and all five nursing outcomes from the NMDS-AT could be extracted from the 20 patient records. Thus, a total of 68 (58.6 %) of the 116 data elements of the NMDS-AT were found in the analysed patient records.

We found that the data needed for the NMDS-AT were partly documented in standardized form (e.g., using a nursing classification systems in nursing care plans) and partly as free text (e.g., in nursing reports, on paper-based fever charts, or in specific electronic forms) (see Table 2).

**Table 2:** Forms and location of nursing data needed for the NMDS-AT in the analysed patient records (*n* = 20)

	Form of documentation		Location in patient record
Nursing diagnoses	nursing classification system	electronic	nursing care plan
Nursing interventions	nursing classification system or free text notes	electronic or paper-based	nursing care plan, nursing report, specific forms or fever chart
Nursing outcomes	check boxes or free text notes	electronic or paper-based	nursing assessment, specific forms, discharge letter or fever chart

The extraction of nursing interventions was the most time-consuming part, because of the different locations and forms of documentation. The nursing outcomes were easier to extract, even if the data could also be found in different locations, because nursing outcomes are always documented on one specific form. Some information (e.g., on patient falls, malnutrition) is always documented in a specific documentation form and thus easy to locate. In contrast, other information (e.g., on cases of restraining patients) is always documented on fever charts, although there sometimes are additional details in the nursing reports. Information about pressure ulcers can either be found in the nursing assessment form, the wound care documentation, the nursing discharge letter, or in the MBDS.

Summarizing, the main challenges for data availability were:

- various locations for documentation, such as nursing care plans, nursing reports, specific forms, or fever charts, with sometimes overlapping information;
- mix of standardized and free text notes in the patient record; and
- mix of paper-based and electronic records, with sometimes overlapping and/or inconsistent information.

This level of unstandardized and overlapping documentation impedes an automatic extraction of NMDS-AT data from the available patient records at the moment.

### Interrater reliability

Three of the thirteen nursing diagnoses, thirteen of the 50 nursing interventions and 100 % of the five nursing outcomes could be extracted with 100 % agreement between both raters on the NMDS-AT form. For extraction of nursing diagnosis, both raters showed agreement of 85 % of higher, with kappa values between 0.35 and 1.0 (Table 3).

**Table 3:** Percentage of agreement (%) and kappa values ($\widehat{\kappa}$) of extracting nursing diagnoses to the NMDS-AT (2 raters, 20 patient records)

NMDS-AT data elements: nursing diagnosis	Interrater/*n* = 20	
	%	$\widehat{\kappa}$
Mobility: Impaired	100	1.00
Transfer Ability and Walking: Impaired	100	1.00
Risk for Fall	100	1.00
Bowel Elimination: Impaired	90	0.46
Urinary Elimination: Impaired	90	0.46
Urinary Incontinence	100	1.00
Bowel Incontinence	100	1.00
Self-Care Deficit: Dressing/Grooming	100	1.00
Self-Care Deficit: Bathing/Hygiene	100	1.00
Self-Care Deficit: Feeding	100	1.00
Self-Care Deficit: Toileting	85	0.35
Risk for Infection	100	1.00
Skin Integrity: Risk for Impaired and Impaired	100	1.00

The three nursing diagnoses ‘Self-Care Deficit: Toileting’ , ‘Bowel Elimination: Impaired’ , and ‘Urinary Elimination: Impaired’ showed a low $\widehat{\kappa}$ ≤0.46. Reasons for missing agreement were:

- Nursing diagnoses, such as ‘Bowel Elimination’ and ‘Urinary Elimination’ were not always correctly used by nurses in the patient record.
- The nursing diagnosis ‘Self-Care Deficit: Toileting’ was often inconsistently documented in the patient record.
- Nurses often make no precise distinctions between ‘Urinary Elimination’ , ‘Bowel Elimination’ and ‘Self-Care Deficit: Toileting’; they often documented ‘Urinary Elimination’ , even if the right nursing diagnosis would have been ‘Self-Care Deficit: Toileting’.

Therefore, these three nursing diagnoses could not be exactly assigned from the nursing documentation to the NMDS-AT form and these nursing diagnoses have poor kappa values.

The interrater reliability for the 50 nursing interventions (*n* = 20) showed ICCs ranging from 0.03 for the nursing intervention ‘Observing Bowel Continence’ to 1.00 for 37 (74 %) nursing interventions (see Table 4).

**Table 4:** Intraclass correlation coefficients of extracting nursing interventions to the NMDS-AT with ICC <1.00 (2 raters, 20 patient records)

NMDS-AT data elements: nursing interventions	ICC
Mobility in the Ward: Take over	0.51
Mobility in the Ward: Assisting	0.95
Care for Elimination: Observing Bowel Continence	0.03
Care for Elimination: Assisting Bowel Continence	0.19
Bowel Management	0.98
Hygiene Care: Prepare	0.62
Hygiene Care: Assisting	0.97
Hygiene Care: Resident	0.67
Wound Care: Surgical/Drains	0.66
Wound Care: Simple	0.99
Wound Care: Complex	0.98
Registration of Vital and Physiological Signs	0.87
Give Information: Unplanned	0.99
Nursing Evaluation	0.95
Multidisciplinary Meeting	0.99
Discharge Management	0.66

Seven of the 50 nursing interventions showed an ICC ≤ 0.75. Reasons for missing agreement were:

- Nurses documented nursing interventions in different ways. For example, ‘Elimination’ was documented either in the nursing documentation or on the fever chart.
- Co-responsible interventions, such as ‘Wound Care: Surgical/Drains’ , are often documented at different locations of the patient record.
- Some nursing interventions, such as ‘Discharge Management’ are usually performed by different professional groups, for example by social workers or nurses with a special skill mix. These nursing activities are also often documented in different ways, such as in the nursing report, in the nursing action plan, or in an additional document.

If nursing interventions are documented in different locations, then the raters may count different frequencies.

All the nursing outcomes were extracted from patient records and all analysed aspects indicated an ICC of 1.00. This can be explained by the fact that all outcome indicators are always documented on one specific form.

### Test-retest reliability

Five patient records were again extracted to assess the test-retest reliability after three months by one of the two raters. The test-retest correlation for the eight extracted nursing diagnoses, 28 nursing interventions, and four nursing outcomes showed 100 % agreement.

### Usefulness of the NMDS-AT

To assess usefulness, we performed selected data analyses on the extracted NMDS-AT data. These data analyses reflected typical analysis questions for an NMDS by clinical managers, policy makers, and researchers. Results are presented in the following paragraphs.

**Fig. 2:**
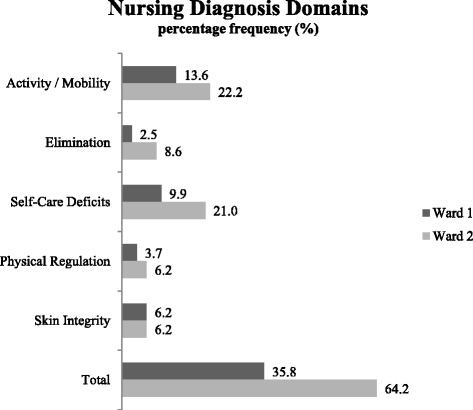
Example of analysis of nursing diagnosis. Comparison of mean percentage frequencies (%) of the ‘Nursing Diagnosis Domains’ on Ward 1 (rehabilitation/aftercare ward) and Ward 2 (rehabilitation/geriatric ward) (*n* = 20 patients)

**Fig. 3:**
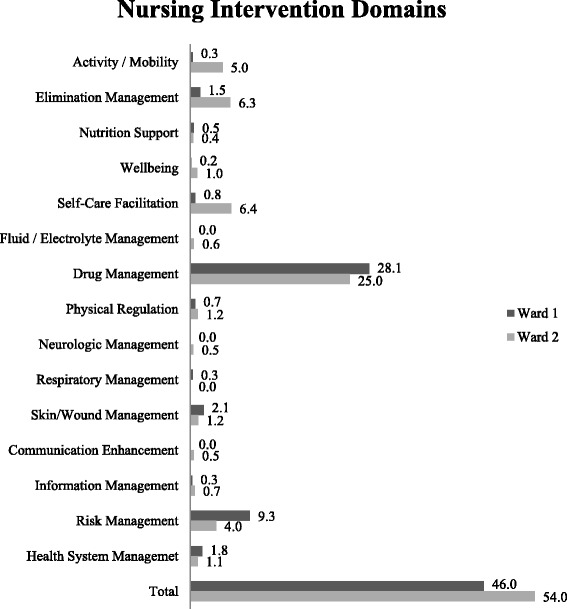
Example of analysis of nursing interventions. Comparison of mean percentage frequencies (%) of the ‘Nursing Intervention Domains’ on Ward 1 (rehabilitation/aftercare ward) and Ward 2 (rehabilitation/geriatric ward) (*n* = 20 patients)

**Fig. 4:**
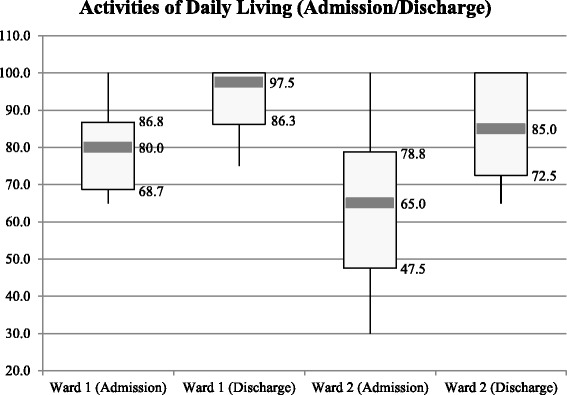
Example of analysis nursing’s contribution. Comparison the mean score of the Barthel Index at admission and discharge on Ward 1 (rehabilitation/aftercare ward) and Ward 2 (rehabilitation/geriatric ward) (*n* = 20 patients)

**Fig. 5:**
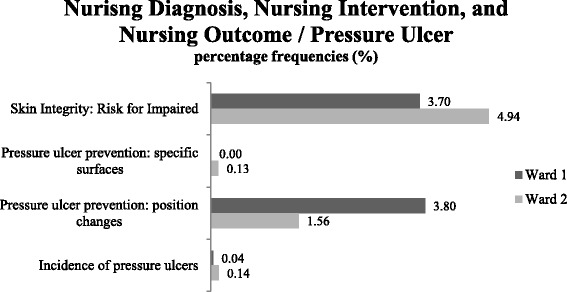
Example of analysis of nursing diagnoses, nursing interventions, and nursing outcomes. Comparison of mean percentage frequencies (%) regarding pressure ulcers on Ward 1 (rehabilitation/aftercare ward) and Ward 2 (rehabilitation/geriatric ward) (*n* = 20 patients). It does not show a causal relationship, merely the flow of events in time

1. Is it possible to describe the diversity of nursing care?
  Frequencies of nursing diagnoses, nursing interventions, and results of nursing care were calculated for both wards (see Figs. 2 and 3). The examples show that diversity between wards and institutions can be represented based on NMDS-AT, this containing useful information for clinical management.

(2) Is it possible to measure nursing care intensity (nursing workload) to support human resource planning and distribution of funds?
  As Fig. 3 shows, percentage frequencies of nursing interventions can visualize nursing care intensity. However, nursing workload cannot be derived directly from this NMDS-AT information without additional workload measurement instruments. Currently, based on the NMDS-AT, it is not possible to support the distribution of funds, as actual costs per nursing intervention have to be available for this.

(3) Is it possible to illustrate nursing’s contribution to patient care to provide arguments for healthcare decision makers?
  Some information from the NMDS-AT can be used to reflect the contribution of nursing care to the healing process of the patient. Our example analysis shows that the Barthel Index rises between admission and discharge (a high Barthel Index shows a high self-employment of the patient) on both wards (Fig. 4). This and comparable analysis from the NMDS-AT may give clinical managers as well as policy makers some arguments on future strategic developments, for example expansion of rehabilitation wards.

(4) Is it possible to evaluate nursing outcomes to support quality management and to improve patient safety, and to identify evidence-based ‘best practice’?
  The NMDS-AT contains nursing outcome indicators. Figure 5 presents an example of their analysis regarding pressure ulcers. This information is important for clinical management and may be used to support quality assurance of nursing care. By comparing wards or institutions, evidence-based ‘best practice’ may also be identified.

(5) Is it possible to describe nursing care for benchmark activities?
  Benchmarking comprises a standardized and quantitative comparison between institutions. Figures 2, 3, 4 and 5 already contained benchmarking examples related to nursing diagnoses, nursing interventions, and nursing outcomes. This helps clinical managers to compare wards or institutions, identify strengths and weaknesses within organisations, identify the level of performance possible by looking at the performance of others, and promote changes. In this study, only two wards were compared, but by using uniformly extracted data, as designed in the NMDS-AT, national benchmarking of nursing care will also be possible.

(6) Is it possible to report frequencies over long time periods to show trends in nursing care?
  This question cannot be answered in this study, because data was not extracted over a longer period of time. In principal, a regular application of the NMDS-AT (e.g., for selected patients every two months) would allow a time-related analysis of all presented analysis questions.

## Discussion

Given the need for systematic description of nursing care, establishing an NMDS-AT and assessing its feasibility is important for the further development of nursing care in Austria. In the 20 patient records, a total of 68 (58.6 %) data elements from the NMDS-AT could be found: 13 nursing diagnoses, 50 nursing interventions, and all five nursing outcomes. Around 41.4 % of the nursing data elements could not be found in these 20 patient records, because only two units with medical and surgical patients were included in this study. Nevertheless, nearly two-thirds of data items could be found and the NMDS-AT thus be tested. Still, future studies in a larger and broader setting are necessary.

Several challenges concerning data extraction were found, including various forms and locations of nursing documentation, such as standardized documentation versus free text notes, and paper-based fever charts versus electronic nursing records. These problems of distributed and unstructured nursing documentation also exist in other Austrian hospitals [32] and in other countries [33]. Thus, currently no automated NMDS-AT analysis seems possible and data extraction for the NMDS-AT must be done manually.

Analysis of reliability of an NMDS is an important indication for the quality of an NMDS and has also been conducted for other NMDS, e.g., the Dutch NMDS. The results of our reliability analysis indicated that ten extracted nursing diagnoses and 43 (86 %) extracted nursing interventions have sufficient interrater reliability ($\widehat{\kappa}$ ≥ 0.61, ICC ≥ 0.75). The intrarater reliability of the five repeated measurements has an overall percentage agreement of 100 %. These results suggest that the NMDS-AT can be reliably applied. Based on the study results, the following modifications of the NMDS-AT were conducted:

- the nursing diagnoses ‘Bowel Elimination: Impaired’ and ‘Urinary Elimination: Impaired’ are polled to the nursing diagnosis ‘Elimination: Impaired’; and
- the nursing interventions ‘Care for Elimination: Observing Bowel Continence’ and ‘Care for Elimination: Assisting Bowel Continence’ are polled to the nursing intervention ‘Care for Elimination’.

Our analysis of the extracted data indicate that NMDS-AT will be able:

- to visualize diversity of nursing care,
- to illustrate benefits of nursing care professionals,
- to support quality assurance as well as to improve patient safety,
- to identify evidence-based ‘best practice’ , and
- to describe nursing care for benchmark activities.

NMDS from other countries have also been used to answer comparable analysis questions, such as to illustrate the differences in patient populations and variations in nursing activities [34], to describe the frequency of intravenous medications [35], to support the implementation of measures regarding quality assurance and patient safety [36], or to describe the characteristics of hospitalized older patients with dementia [37].

NMDS-AT is not able at the moment to answer questions regarding nursing workload, distribution of funds, and trend analyses in nursing care practice. Other NMDS studies addressing nursing workload based on the NMDS [38, 39] show the need to integrate a nursing workload measurement system, such as the San Joaquin patient classification system in the B-NMDS [39, 40]; this is not the case in the NMDS-AT at the moment. Using NMDS-AT data to distribute funds would need to include recent developments of nursing cost-weights per DRG [41] and nursing related groups (NRGs) based on the B-NMDS [42].

In this study, ‘Data of the institution’ , ‘Patient demographics’ and ‘Medical care elements’ were extracted manually from patient charts. However, these data are also included in the MBDS, which is already recorded electronically for DRG-related data reporting to the Federal Ministry of Health [24]. Using a unique patient code, this MBDS data could be automatically linked to the NMDS-AT in the future [5, 43].

### Strengths and limitations

Overall, our study found that NMDS-AT data elements deliver reliable and valid information about nursing care, even if the study has some limitations: First, the NMDS-AT was tested in one hospital only, so the included hospital cannot represent all possible acute care settings. Second, a total of 457 patient days (20 included patients) is a small sample, although Charter and Feldt [44] argue that ‘it is not theoretically defensible to set a universal standard for test score reliability’. Third, while interrater and intrarater reliability is an important element in reliability testing of an instrument, it should be noted that this is only one of several reliability indicators [45]. However, the aim of this study is to examine the feasibility of the NMDS-AT as a data extraction instrument as well. In this context, the reliability test evaluates the objectivity and stability of the NMDS-AT. Fourth, the quality and content of nursing documentation will differ across organisations and settings. Therefore, this study might have yielded different results in other organisations or settings. However, in Austria, compared with other countries [46–48], the nursing diagnoses are implemented in a more uniform way due to mandatory legislative requirements (GuKG, 1997) [16]. Before broader implementation of NMDS-AT in another hospital, the NMDS-AT should be tested in paper-based form to test data availability and extraction guidelines.

### Challenges of NMDS-AT implementation

The NMDS-AT data are available in the patient record and the extracted data are useful. For the implementation of the NMDS-AT, however, some challenges need to be addressed:

First, nurses should be instructed about the importance of nursing documentation and should be informed why providing nursing data is necessary. For example, in the categories ‘Mobility: Impaired’ and ‘Self-Care Deficits’ , it is commonly unclear for nurses which nursing diagnoses are represented in an actual patient situation in order to ensure best patient care. Comparisons of nursing data are complicated if nurses use different reasoning regarding nursing diagnosis [49]. Theoretically, there are indeed guidelines for good decision making in diagnostics; in practice, however, nurses are required to make rapid choices. An important step in supporting nurses is to educate them regarding clinical decision making (e.g., [50, 51]), to develop guidelines for good decision making regarding nursing diagnosis, and to provide standards for hospital information systems in view of the fact that secondary data analysis is becoming increasingly important in healthcare.

Second, the level of unstandardized and overlapping documentation in routine nursing documentation in many Austrian hospitals makes automated data extraction for the NMDS-AT very difficult. This also is a known problem in other countries [52]. Optimally, integrated electronic solutions for patient records should be used in the future. Standards pertaining to electronic healthcare systems – supporting the documentation of nursing practice, on the one hand, and secondary data analysis [53], on the other hand – should be developed.

Third, the documentation of nursing interventions as free text in nursing reports may cause a loss of essential information for data extraction. In order to allow automated data extraction in the future, and to obtain comparable data, standardized documentation of nursing interventions is necessary. In other NMDS such as the B-NMDS II, these problems have not been reported [54], because nurses uniformly seem to use the nursing interventions codes from B-NMDS II to document their intervention in the patient record.

Fourth, there was also some concern about how nursing data in the patient record can be mapped to the NMDS-AT. The locally developed nursing classification system in the patient record of the observed hospital is used directly by nurses to record nursing diagnoses and nursing interventions. In our study, we identified some challenges to mapping the nursing classification system in the patient record on the NMDS-AT data: the availability of actual information to assess the quality and completeness of terminology linkage; the difficulty of correctly using classifications systems; and the need to address differences in granularity between both terminologies. These results are similar to experiences of other mappings [55]. During the implementation of NMDS-AT in a hospital, a manual approach, as was used in this study, can be recommended to allow detection and discussion of possible discrepancies between mappings of different nursing classification systems to the NMDS-AT. Therefore, the nursing classification system of the patient record should be mapped to the NMDS-AT by using the approach described in ISO 25964-2: 2013 [56]. Nevertheless, for future data extractions, an automated mapping will be necessary, for example by using the Unified Medical Language System (UMLS) of the US National Library of Medicine (NLM) [57]. The linkage of the nursing outcomes from the patient record to the NMDS-AT proved to be more complicated, because some data elements of the nursing outcomes are measured by scores, such as the Barthel Index of Activities of Daily Living. If an analysis of nursing outcomes is planned, it is necessary to use validated standardized measurement instruments. However, different standardized measurement instruments were used in clinical practice. As part of data extraction, the name of the measurement instruments used should be documented to allow comparison of data from the same measurement instrument.

Fifth, ethical considerations are very important [58]. In this study, the privacy and security of personal data were repeatedly emphasized. Privacy and security of personal data in data sets must be paramount. Before implementation of the NMDS-AT, a detailed data protection concept must be created. This concept must be geared to regional/institutional and national frameworks.

### Future development of the NMDS-AT

The proposed NMDS-AT focuses on the long-term and inpatient care setting. If the NMDS-AT is used with inpatients with a short length of stay, it is expected that the NMDS-AT would measure similar results. The NMDS-AT does not consider paediatrics, maternity, psychiatric speciality, or outpatient settings at the moment, but developments in this direction are planned.

For the future, it is planned to test the NMDS-AT in further healthcare institutions. In addition, the NMDS-AT will be mapped with a nursing workload measurement system. It would also be interesting to map the NMDS-AT with the International Classification for Nursing Practice (ICNP®) as reference terminology, such as NR Hardiker, W Sermeus and K Jansen [59] show.

Considering future developments of an electronic health record system (ELGA) in Austria [60], the contents of ELGA should be geared to the NMDS-AT. It would also be interesting to see whether data from ELGA can be used for the NMDS-AT and how to support the linkage of different healthcare data sets by using a Master Patient Index.

For a national introduction of the NMDS-AT, it is indispensable to regulate the NMDS strategy by law, as experience in Belgium has shown [61].

## Conclusions

The NMDS-AT shows good data availability, reliability, and usefulness to support clinical managers, policy makers, and nursing researchers. But before the NMDS-AT can be introduced in healthcare institutions, some challenges need to be addressed: 1. improving the quality of nursing documentation; 2. reducing fragmentation of documentation; 3. using a standardized nursing classification system; and 4. mapping between the nursing classification system in EHR and the NMDS-AT.
