# Erratum to: Implementation of the Austrian Nursing Minimum Data Set (NMDS-AT): A Feasibility Study

**DOI:** 10.1186/s12911-015-0238-3

**Published:** 2015-12-30

**Authors:** Renate Ranegger, Werner O. Hackl, Elske Ammenwerth

**Affiliations:** Steiermärkische Krankenanstaltengesellschaft m.b.H., Management/Pflege, Stiftingtalstraße 4-6, Graz, 8010 Austria; Institute of Biomedical Informatics, UMIT -University for Health Sciences, Medical Informatics and Technology, Eduard Wallnöfer-Zentrum I, Hall in Tirol, 6060 Austria

After the publication of this article [1] it was noticed by the author that the article contained a couple of errors. On page three, under the sub-heading ‘Standardized form for data extraction’ it is stated that the form contained 6 nursing outcomes, whereas this should read as 5 nursing outcomes. Also, on page four, under the sub-heading ‘Data analysis’ there are two mistakes which refer to interclass. However, this should read as intraclass.
